# Atherosclerosis and Its Impact on the Outcomes of Patients with Deep Venous Thrombosis

**DOI:** 10.3390/life12050734

**Published:** 2022-05-14

**Authors:** Karsten Keller, Jürgen H. Prochaska, Meike Coldewey, Sebastian Göbel, Volker H. Schmitt, Omar Hahad, Alexander Ullmann, Markus Nagler, Heidrun Lamparter, Christine Espinola-Klein, Thomas Münzel, Philipp S. Wild

**Affiliations:** 1 Department of Cardiology, University Medical Center Mainz of Johannes Gutenberg-University Mainz, 55131 Mainz, Germany; meikeco@googlemail.com (M.C.); sebastian.goebel@unimedizin-mainz.de (S.G.); volker.schmitt@unimedizin-mainz.de (V.H.S.); omar.hahad@unimedizin-mainz.de (O.H.); espinola@uni-mainz.de (C.E.-K.); tmuenzel@uni-mainz.de (T.M.); 2 Center for Thrombosis and Hemostasis, University Medical Center Mainz of Johannes Gutenberg-University Mainz, 55131 Mainz, Germany; juergen.prochaska@unimedizin-mainz.de (J.H.P.); steinforter@googlemail.com (A.U.); markus.nagler@unimedizin-mainz.de (M.N.); heidrun.lamparter@unimedizin-mainz.de (H.L.); philipp.wild@unimedizin-mainz.de (P.S.W.); 3 Department of Sports Medicine, Medical Clinic VII, University Hospital Heidelberg, 69120 Heidelberg, Germany; 4 Preventive Cardiology and Preventive Medicine, Department of Cardiology, University Medical Center of Johannes Gutenberg-University Mainz, 55131 Mainz, Germany; 5 German Center for Cardiovascular Research (DZHK), Partner Site Rhein Main, 55131 Mainz, Germany; 6 Leibniz Institute for Resilience Research (LIR), 55131 Mainz, Germany

**Keywords:** deep vein thrombosis, pulmonary embolism, atherosclerosis, peripheral artery disease, diabetes, age

## Abstract

Introduction: Atherosclerosis and pulmonary embolism (PE) affect cardiovascular mortality substantially. We aimed to investigate the impact of atherosclerosis on the outcomes of patients with deep venous thrombosis (DVT) and to identify the differences in DVT patients with and without PE. Methods: Patients with DVT with and without symptomatic atherosclerosis (defined as coronary artery disease, myocardial infarction and/or peripheral artery disease) as well as with and without PE under oral anticoagulation were enrolled during January 2011–April 2013 and compared. The impact of symptomatic atherosclerosis on several outcomes was analyzed. Results: Overall, 509 DVT patients (70.0 [56.0–77.0] years, 51.9% females) were included in this study. Among them, 179 (36.3%) had symptomatic atherosclerosis and 204 (40.1%) a concomitant PE. DVT patients with symptomatic atherosclerosis were older (74.0 [IQR 65.0–80.0] vs. 63.0 [48.0–75.0] years, *p* < 0.0001), more often male (56.4% vs. 43.9%, *p* = 0.0087) and had a higher prevalence of classical CVRF and a higher Charlson comorbidity index (7.00 [5.00–8.00] vs. 4.00 [2.00–6.00], *p* < 0.001). Symptomatic atherosclerosis was associated with increased mortality (HR 1.98 [95%CI 1.12–3.49], *p* = 0.018) and hospitalizations (HR 1.64 [95%CI 1.21–2.21], *p* = 0.0012) and primary long-term outcome (HR 1.99 [95%CI 1.31–3.04], *p* = 0.0013) during the 2 years follow-up-period in DVT patients. DVT patients without PE had diabetes mellitus (28.2% vs. 16.3%, *p* < 0.01) and symptomatic atherosclerosis (42.9% vs. 26.4%, *p* < 0.001) more often compared to DVT patients with PE, and symptomatic atherosclerosis was associated with isolated DVT (without PE) (OR 2.01 [95%CI 1.28–3.16], *p* < 0.01). Conclusions: Atherosclerosis was associated with isolated DVT (without PE) and increased mortality in DVT patients under oral anticoagulation. The profile of CVRF and comorbidities differed between DVT patients with and without a concomitant PE. In the case of DVT or PE, patients should be screened for concomitant atherosclerotic disease. Clinical Trial Registration: at clinicaltrials with Unique identifier NCT01809015.

## 1. Introduction

Deep venous thrombosis (DVT) and pulmonary embolism (PE) are the two clinical presentations of venous thromboembolism (VTE). The incidence of DVT is estimated at 44–145 per 100,000 individuals in the general population [1,2,3,4,5]. VTE constitutes a major global burden of disease with significant morbidity and mortality [5,6,7]. Current international guidelines recommend anticoagulant treatment for at least 3 months aiming to treat the current VTE event as well as prevent recurrent VTE events [5,7,8,9,10]. Although VTE events are frequently related to important risk factors such as trauma, immobilization, surgery, high age, myocardial infarction, heart failure, thrombophilia and cancer [11,12,13,14,15,16,17,18,19], a cumulating body of evidence suggests that VTE risk factors might not be equally important in both VTE entities. Cancer and thrombophilia are more prevalent in DVT, whereas major surgery and trauma, high age, myocardial infarction, atrial fibrillation (AF) and heart failure are predominantly associated with PE [17,19,20,21]. Since approximately 2/3 of all PE events are the consequence of DVT, these VTE cases with PE resulting from DVT are not regarded as a separate clinical entity [3,22]. In contrast, for isolated PE (PE without underlying DVT), study results suggest a key role of co-morbidities such as cancer [23], AF [20,21], myocardial infarction [21] and heart failure [20,21] regarding the pathogenesis of thrombus formation [19].

In recent years, an association between atherosclerosis and DVT was confirmed in several studies [24,25,26,27,28], beginning with the study of Prandoni et al., who reported a higher frequency of carotid plaques as an indicator of atherosclerosis in patients with previous idiopathic DVT in comparison to the controls without DVT [25]. Other studies emphasized that aortic calcifications [29] and also coronary artery calcium were more frequent in patients with VTE in comparison to controls [30]. In two large studies analyzing the German nationwide inpatient sample, we observed a substantial impact of atherosclerosis on short-term in-hospital outcomes of VTE patients [28,31]. Thus, the main objective of the present study was to investigate the impact of symptomatic atherosclerosis on the outcomes of DVT patients.

## 2. Methods

### 2.1. Study Design and Study Sample

The thrombEVAL study (registered at: http://clinicaltrials.gov, unique identifier: NCT01809015, accessed on 12 March 2013) was designed as an observational study to compare the quality of oral anticoagulation with vitamin K antagonist treatment (VKA) between patients treated in general medical care and those treated by a specialized telemedicine-based coagulation service [32,33,34,35]. For the current analysis, all individuals with a history of DVT were analyzed. The DVT patients from both study cohorts were included and stratified regarding a co-prevalence of PE as well as the existence of symptomatic atherosclerosis (defined as the presence of coronary artery disease [CAD], acute myocardial infarction [MI] and/or peripheral artery disease [PAD]). In addition, patients with isolated DVT (without concomitant PE) were compared to those with isolated PE (without concomitant DVT).

A total of 2318 patients were enrolled in 21 study-center hospitals between January 2011 and April 2013. Patients ≥ 18 years of age with an indication for VKA were eligible for participation in this observational study. All of the participants provided written informed consent prior to study enrolment [35]. Inclusion and exclusion criteria are shown in Table S1 in the Supplementary Material.

In both ThrombEVAL study cohorts, the data were accurately assessed at baseline during a clinical visit using medical records and this information, as well as the laboratory data. These data were incorporated and implemented in an electronic case report file (eCRF) with predefined checks for plausibility according to standard operating procedures in both cohorts. Diagnosis and history of PE, as well as DVT, were assessed in the medical report and patients’ anamnesis. Outcome data (i.e., hospitalization, primary long-term outcome, mortality and thromboembolic arterial and venous events under VKA) were assessed and measured via regular endpoint assessment through computer-assisted telephone interviews as well as clinical visits. All data underwent a detailed quality control check before analysis. All information on study endpoints was validated by medical records and adjudicated by an independent review panel. In order to minimize loss to follow-up, checks with registration offices on mortality were performed on a regular basis. Study monitoring was carried out by an independent institution. Further definitions are described in the Supplementary Material.

The rationale and design of the thrombEVAL study was recently described in detail [32]. All procedures followed the principles of good clinical practice as well as Strengthening the Reporting of Observational Studies in Epidemiology (STROBE) guidelines and the Declaration of Helsinki. Local ethics boards and the local data safety commissioner approved the study protocol (medical association Rhine-Hessen, Germany; reference no. 837.407.10.7415/7416). Clinical Trial Registration: URL: http://www.clinicaltrials.gov; unique identifier NCT01809015 (accessed on 12 March 2013).

### 2.2. Study Endpoints

Mortality under VKA comprised deaths of all causes during follow-up. The primary long-term outcome was defined as a combined endpoint of thromboembolic arterial and venous events, major and clinically relevant bleedings and death of all causes under VKA. Hospitalization under VKA comprised all hospital admissions during follow-up. Major bleeding was defined as (i) bleeding events with a reduction in haemoglobin levels of at least 2.0 g/L, (ii) bleeding events leading to the transfusion of at least two units of blood or packed cells or (iii) symptomatic bleeding events in a critical area or organ such as intra-spinal, intra-cranial, intra-ocular, retroperitoneal bleeding, intra-articular bleeding or pericardial bleeding [36].

Clinically relevant non-major bleeding was defined as a bleeding event requiring medical attention in an ambulatory or clinical setting. Clinically relevant bleeding comprised the composite endpoint of major bleeding and clinically relevant non-major bleeding [37]. Thromboembolic arterial and venous events under VKA were defined as a sum of VTE events, MI and stroke/TIA under VKA during follow-up. Recurrent VTE included recurrent DVT or PE.

The follow-up period was determined as two and three years after the inclusion of the individual patient.

### 2.3. Statistical Analysis

The frequencies of classical cardiovascular risk factors (CVRF) and comorbidities in DVT patients with and without concomitant PE, DVT patients with and without symptomatic atherosclerosis, as well as isolated DVT (without concomitant PE) vs. isolated PE (without concomitant DVT) were investigated. Moreover, associations between the DVT groups and CVRF as well as comorbidities were statistically examined.

The descriptive analysis presented dichotomous variables by absolute and relative frequencies, and differences in the proportions were tested using the Fisher’s Exact Test. The median and interquartile range or mean and standard deviation were utilized to describe continuously distributed variables as appropriate. Parameters of the investigated groups were compared with the Mann–Whitney *U*-test if they were not normally distributed or with the unpaired *t*-test if they were normally distributed.

Logistic regression models were analyzed in order to assess associations between isolated DVT (without concomitant PE) and CVRF as well as co-morbidities. All models were presented univariably or multivariably. Sex, age and CVRF were computed as independent predictors for a history of PE in multivariable logistic regression models in patients with DVT: the model contained the following variables: sex, age, diabetes, obesity, arterial hypertension, dyslipidemia, family history of MI or stroke and smoking. In addition, multivariable logistic regression models were used to evaluate the association of comorbidities with a concomitant PE (i.e., the dependent variable; reference: no history of PE). In model 1, each concomitant disease was adjusted for CVRF (i.e., the independent variables) in a separate model, and in model 2, all concomitant diseases (MI was not taken for adjustment due to the co-linearity with CAD and PAD; CAD and MI were not taken for adjustment due to the co-linearity with symptomatic atherosclerosis) and CVRF were included in one model. Furthermore, multivariable logistic regression models were used to evaluate the association between DVT with concomitant PE (i.e., the dependent variable; reference: no history of PE) and the escalation of anti-diabetic treatment under adjustment for cardiovascular risk factors (i.e., the independent variables) and multivariable logistic regression models was used to evaluate the association between a DVT with PE (i.e., the dependent variable; reference: no history of PE) and the sub-classes of obesity while adjusting for CVRF (i.e., the independent variables) in DVT patients. The obesity sub-classes were defined according to the World health Organization (WHO, 2008), which defined obesity class I as a BMI between 30.0 and 34.9 kg/m^2^, class II as a BMI between 35.0–39.9 kg/m^2^ and class III as a BMI ≥ 40.0 kg/m^2^. The adjusted odds ratios (OR) are provided with 95%-confidence intervals and *p*-values. Moreover, CVRF as independent predictors for isolated PE (without concomitant DVT) in multivariable logistic regression models vs. patients with isolated DVT (without concomitant PE as the reference group) were computed (the model contained the following variables: sex, age, diabetes mellitus, obesity, hypertension, dyslipidemia, family history of myocardial infarction or stroke and smoking) and multivariable logistic regression models were used to evaluate the association of comorbidities with isolated PE (without concomitant DVT, i.e., the dependent variable; reference: isolated DVT [without concomitant PE]). In model one, each concomitant disease was adjusted for CVRF (i.e., the independent variables) in a separate model, and in model two, all concomitant diseases were adjusted (myocardial infarction was not taken for adjustment due to the co-linearity with coronary artery disease and peripheral artery disease).

Regarding the endpoints (hospitalizations, mortality, primary long-term outcome, thromboembolic arterial and venous events and recurrent VTE) under VKA treatment, Kaplan–Meier curves were computed for a 2-year follow-up period under treatment. The comparisons of the curves of DVT patients with and without symptomatic atherosclerosis were tested with the log-rank test. Moreover, Cox-regressions for hazard ratios (HR) with 95% CI (denoted in brackets) were calculated in order to analyze the impact of symptomatic atherosclerosis on the outcomes for 2-year and 3-year follow-up. To confirm the crude results of the univariable Cox regressions, we computed a propensity matching of the two DVT groups of patients with and without symptomatic atherosclerosis for age, sex, CVRF and comorbidities including heart failure, chronic obstructive pulmonary disease, renal failure, history of bleeding, AF, cancer and depression) and calculated the Cox regressions once again in the propensity-matched groups to analyze the impact of symptomatic atherosclerosis on the outcomes for 2-year and 3-year follow-up.

A value of *p* < 0.05 was considered a significant association. All tests were carried out two-sided. Analyses were performed with R, Version 3.0.3.

## 3. Results

Overall, 509 patients with a medical history of DVT (median age: 70.0 (56.0–77.0) years, 51.9% females) and 91 patients with isolated PE (without concomitant DVT) were included in the present study. Among these DVT patients, 305 (59.9%) of these DVT patients had an isolated DVT (without concomitant PE), whereas 204 DVT patients (40.1%) had a history of DVT with a concomitant PE.

### 3.1. Comparison of DVT Patients with and without Additional PE: Isolated DVT (without Concomitant PE) Is Accompanied by Unfavorable Cardiovascular Profile

In addition to comparable age and sex-distribution, DVT patients with an isolated DVT (without concomitant PE) suffered from diabetes mellitus (28.2% vs. 16.3%, *p* < 0.01) more often and had a more positive family history of MI or stroke (41.3% vs. 30.4%, *p* = 0.02) (Table 1). Remarkably, atherosclerotic diseases such as acute MI (19.4% vs. 11.8%, *p* = 0.03), CAD (32.3% vs. 17.8%, *p* < 0.001) and PAD (21.3% vs. 10.9%, *p* < 0.01) were far more prevalently found in DVT patients without PE. Consequently, symptomatic atherosclerosis was significantly more common in isolated DVT (without concomitant PE) (42.9% vs. 26.4%, *p* < 0.001) compared to DVT patients with PE. The Charlson comorbidity illustrates the unfavorable comorbidity profile of patients with isolated DVT (without concomitant PE) (5.59 ± 3.10 vs. 4.93 ± 3.05, *p* = 0.02) (Table 1).

**Figure 1:**
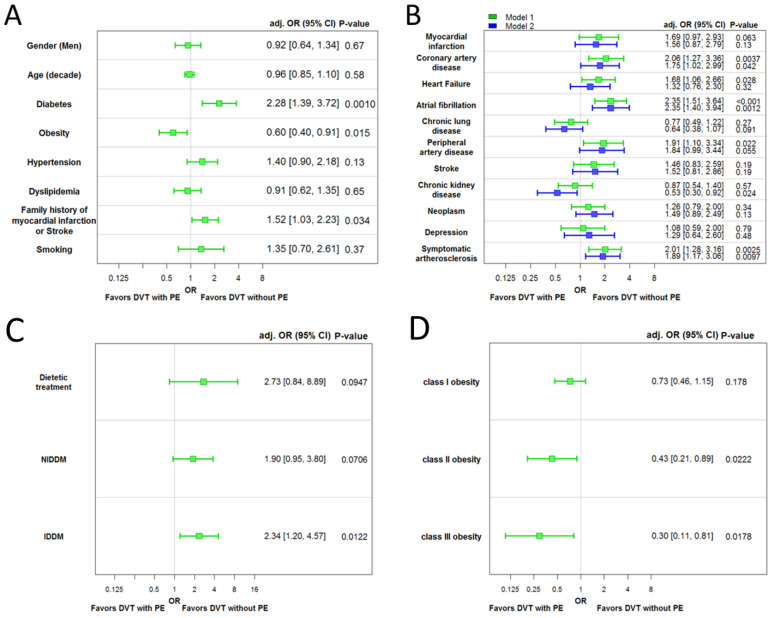
Associations of classical cardiovascular risk factors and comorbidities with DVT with or without PE. (**A**) Cardiovascular risk factors as independent predictors for a history of PE in multivariable logistic regression models in patients with DVT: the model contained the following variables: sex, age, diabetes, obesity, hypertension, dyslipidemia, family history of myocardial infarction or stroke and smoking. (**B**) Multivariable logistic regression models were used to evaluate the association with concomitant history of PE (i.e., the dependent variable; reference: no history of PE). In model 1, each concomitant disease was adjusted for cardiovascular risk factors (i.e., the independent variables) in a separate model, and in model 2, all concomitant diseases (MI was not taken for adjustment due to the co-linearity with CAD and PAD; CAD and MI were not taken for adjustment due to the co-linearity with symptomatic atherosclerosis) and CVRF were included in one model. Symptomatic atherosclerosis was defined as the presence of CAD, MI and/or PAD. (**C**) Multivariable logistic regression models were used to evaluate the association between DVT with concomitant PE (i.e., the dependent variable; reference: no history of PE) and the escalation of anti-diabetic treatment under adjustment for cardiovascular risk factors (i.e., the independent variables). (**D**) Multivariable logistic regression models were used to evaluate the association between a DVT with PE (i.e., the dependent variable; reference: no history of PE) and the sub-classes of obesity while adjusting for cardiovascular risk factors (i.e., the independent variables) in DVT patients. The obesity sub-classes were defined according to the World health Organization (WHO, 2008), which defined obesity class I as BMI a between 30.0 and 34.9 kg/m^2^, class II as a BMI between 35.0–39.9 kg/m^2^ and class III as a BMI ≥ 40.0 kg/m^2^. Abbreviations: BMI = body mass index; DVT = deep venous thrombosis; PE = pulmonary embolism; NIDDM = non-insulin-dependent diabetes mellitus; IDDM = insulin-dependent diabetes mellitus. *p*-values < 0.05 were considered as significant associations.

Multivariable logistic regression models analyzing the association of CVRF as well as comorbidities with isolated DVT (without concomitant PE) vs. DVT with PE as a reference group identified diabetes mellitus (OR 2.28 [95%CI 1.39–3.72], *p* < 0.01) and family history of MI or stroke (OR 1.52 [95%CI 1.03–2.23], *p* = 0.03) as independently associated with isolated DVT (without concomitant PE), whereas obesity (OR 0.60 [95%CI 0.40–0.91], *p* = 0.02) was significantly associated with DVT in co-prevalence with PE (Figure 1A). Regarding comorbidities, CAD (OR 2.06 [95%CI 1.27–3.36], *p* < 0.01), PAD (OR 1.91 [95%CI 1.10–3.34], *p* < 0.05) and symptomatic atherosclerosis (OR 2.01 [95%CI 1.28–3.16], *p* < 0.01) as well as HF (OR 1.68 [95%CI 1.06–2.66], *p* < 0.05) and AF (OR 2.35 [95%CI 1.51–3.64], *p* < 0.001) were associated with isolated DVT (without concomitant PE) independently of age, sex and CVRF (model 1 in Figure 1B). After additional adjustment for comorbidities, the associations remained independent for CAD, AF and symptomatic atherosclerosis. After this further adjustment, chronic kidney disease revealed a significant association with PE in DVT patients (model 2 in Figure 1B).

Severe diabetes mellitus (insulin-dependent diabetes mellitus) was strongly associated with isolated DVT (without concomitant PE) (Figure 1C), whereas obesity classes II and III were associated with PE in DVT patients (Figure 1D).

### 3.2. Comparison of Patients with Isolated Deep Venous Thrombosis (without Concomitant Pulmonary Embolism) vs. Patients with Isolated Pulmonary Embolism (without Concomitant Deep Venous Thrombosis)

Overall, 305 patients with isolated DVT (without concomitant PE) and 91 with isolated PE (without concomitant DVT) were included in this additional analysis of the present study (Table S2 in the Supplementary Material). Patients with isolated PE (without concomitant DVT) were distinctly older than patients with isolated DVT (without concomitant PE) (74.0 [68.0/80.0] vs. 70.0 [57.0/78.3] years, *p* = 0.0012) and more often had AF (40.1% vs. 53.8%, *p* = 0.022) as well as chronic lung diseases (19.5% vs. 37.8%, *p* = 0.00063). In contrast, PAD was more prevalent in isolated DVT (without concomitant PE) in comparison to isolated PE (without concomitant DVT) (21.3% vs. 10.2%, *p* = 0.020) (Table S2 in the Supplementary Material). The proportion of symptomatic atherosclerosis in patients with isolated DVT (without concomitant PE) vs. patients with isolated PE (without concomitant DVT) was not different (42.9% vs. 44.9%, *p* = 0.81).

The multivariate regression model confirmed an independent association between older age (OR 1.28 [95%CI 1.05–1.57], *p* = 0.016) as well as chronic lung diseases (OR 2.43 [95%CI 1.32–4.48], *p* = 0.004) with isolated PE (without concomitant DVT), whereas PAD was associated with isolated DVT (without concomitant PE) (OR 0.36 [95%CI 0.15–0.85], *p* = 0.021) (Figure S2 in the Supplementary Material). Symptomatic atherosclerosis was not independently associated with one of the groups (OR 0.07 [95%CI 0.05–7.51], *p* = 0.707).

### 3.3. Baseline Comparison between DVT Patients with and without Symptomatic Atherosclerosis

Overall, 493 patients with a medical history of DVT (median: 69.0 (55.7–77.0) years, 51.5% females) were included for analysis regarding the comparison of DVT patients with and without symptomatic atherosclerosis. Among them, 179 patients (36.3%) had a co-prevalence of DVT with symptomatic atherosclerosis (defined as the presence of CAD, acute MI and/or PAD) and 314 DVT patients (63.7%) presented without symptomatic atherosclerosis (Table 2).

DVT patients with symptomatic atherosclerosis were distinctly older (median: 74.0 [IQR 65.0–80.0] vs. 63.0 [48.0–75.0] years, *p* < 0.0001), more often of male sex (56.4% vs. 43.9%, *p* = 0.0087) and revealed a higher prevalence of classical CVRF (Table 2). In total, 46.4% of the patients with symptomatic atherosclerosis suffered from acute MI, 75.9% had CAD and in 48.3% a PAD was present. Additionally, the atherosclerosis associated diseases HF (41.2% vs. 14.4%, *p* < 0.0001) and AF (48.3% vs. 23.2%, *p* < 0.0001) were more frequently present in DVT patients with symptomatic atherosclerosis. The Charlson comorbidity index (7.00 [5.00–8.00] vs. 4.00 [2.00–6.00], *p* < 0.001) was higher in patients with symptomatic atherosclerosis (Table 2).

### 3.4. Impact of Symptomatic Atherosclerosis on Outcomes in Patients with Deep Venous Thrombosis

**Figure 2:**
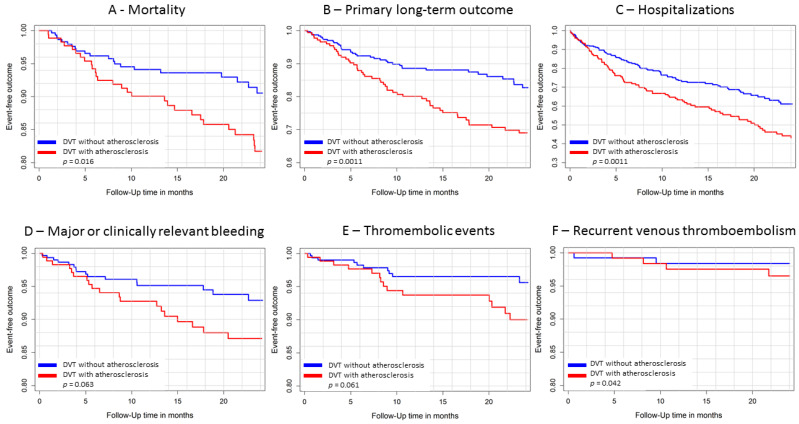
Comparison of DVT patients with and without additional symptomatic atherosclerosis regardless of the presence of additional PE during a 2-year follow-up period under VKA treatment. (**A**) Kaplan–Meier-curve for mortality of DVT patients with and without symptomatic atherosclerosis. (**B**) Kaplan–Meier-curve for primary long-term outcome of DVT patients with and without symptomatic atherosclerosis. (**C**) Kaplan–Meier-curve for hospitalizations of DVT patients with and without symptomatic atherosclerosis. (**D**) Kaplan–Meier-curve for major or clinically relevant bleeding of DVT patients with and without symptomatic atherosclerosis. (**E**) Kaplan–Meier-curve for thromboembolic arterial and venous events of DVT patients with and without symptomatic atherosclerosis. (**F**) Kaplan–Meier-curve for recurrent VTE of DVT patients with and without symptomatic atherosclerosis. Abbreviations: DVT = deep venous thrombosis; PE = pulmonary embolism. Differences in Kaplan–Meier curves were tested with the log-rank test. *p*-values < 0.05 were considered as significant associations.

The Kaplan–Meier curves with log-rank tests revealed significant differences between the groups regarding mortality, hospitalizations and primary long-term outcomes during 2 years of follow-up (Figure 2). In accordance with these results, symptomatic atherosclerosis was associated with increased all-cause mortality (HR 1.98 [95%CI 1.12–3.49], *p* = 0.018), hospitalizations (HR 1.64 [95%CI 1.21–2.21], *p* = 0.0012 and the primary long-term outcome (HR 1.99 [95%CI 1.31–3.04], *p* = 0.0013) in the crude univariate Cox regressions after 2 years follow-up. These results remained stable after 3 years of follow-up (Table 3). After propensity matching of the DVT patient-groups with and without symptomatic atherosclerosis for age, sex, CVRF and comorbidities, symptomatic atherosclerosis in DVT patients affected the primary long-term outcome (HR 1.21 [95%CI 1.02–1.44], *p* = 0.030), all-cause mortality (HR 1.31 [95%CI 1.03–1.66], *p* = 0.027) and thromboembolic arterial and venous events (HR 2.18 [95%CI 1.45–3.28], *p* = 0.00018) significantly after 2 years-follow-up, but not recurrent VTE (HR 2.02 [95%CI 0.96–4.23], *p* = 0.063) and hospitalizations (HR 1.07 [95%CI 0.78–1.48], *p* = 0.66). Analyzing the 3-year follow-up period after propensity matching, systematic atherosclerosis still influenced the primary long-term outcome (HR 1.22 [95%CI 1.05–1.43], *p* = 0.010), all-cause mortality (HR 1.24 [95%CI 1.01–1.51], *p* = 0.038) and thromboembolic arterial and venous events (HR 1.66 [95%CI 1.19–2.31], *p* = 0.0026) significantly, but still not recurrent VTE (HR 0.91 [95%CI 0.54–1.53], *p* = 0.72) and hospitalizations (HR 1.04 [95%CI 0.78–1.40], *p* = 0.79).

## 4. Discussion

The ThrombEVAL trial comprised 509 patients with DVT and 91 patients with isolated PE (without concomitant DVT), providing important insights regarding differences in the importance of the cardio-vascular profile in DVT and PE patients.

The main results of our study can be summarized as follows:

(i) Isolated DVT (without concomitant PE) was accompanied by a substantially higher prevalence of symptomatic atherosclerosis and related diseases in comparison to DVT patients with PE;
(ii) Diabetes mellitus, a key player in the development of atherosclerosis, might also have a key role in DVT patients without PE;
(iii) The prevalence of PAD was substantially higher in patients with isolated DVT (without concomitant PE) in comparison to isolated PE (without concomitant DVT);
(iv) Obesity seems to be an independent risk factor for the development of PE in DVT patients;
(v) Isolated PE (without concomitant DVT) was associated with old age, chronic lung diseases and AF;
(vi) Symptomatic atherosclerosis affected mortality and thromboembolic arterial and venous events in DVT patients under VKA treatment during the 2-year and 3-year follow-up period.

Although VTE and thromboembolic arterial disease are generally considered different disease entities, an increased burden of evidence underlines a potential link between venous and arterial thrombosis. Several studies showed associations between VTE and atherosclerosis in recent decades [13,16,26,28,31,39,40,41,42,43,44,45,46,47,48]. Prandoni et al. reported in their very important study a higher prevalence of atherosclerotic lesions in patients with idiopathic DVT than in DVT patients with secondary DVT as well as controls without DVT [25]. Following conducted studies demonstrated that aortic calcifications [29] and coronary artery calcium were more frequent in patients with VTE in comparison to controls [30]. In addition, a higher prevalence regarding the occurrence of DVT events was detected in patients with PAD than in controls without PAD [27,31].

The prevalence of atherosclerotic diseases in our study was higher than in the published data for the general population [49]. While the frequency of symptomatic atherosclerosis in DVT patients with PE (26.4%) was similar to or only slightly higher than the prevalence of atherosclerotic diseases in the general population, isolated DVT (without concomitant PE) was accompanied by a substantially higher prevalence of symptomatic atherosclerosis (42.9%) compared to the same age group of the general population [49]. The association between isolated DVT (without concomitant PE) and atherosclerosis was distinctly stronger than that between atherosclerosis and DVT with a concomitant PE. Diabetes mellitus as a key player in the development of atherosclerosis and its complications [50] was also strongly and independently associated with isolated DVT (without concomitant PE) in comparison to DVT with PE. In contrast, obesity was associated with PE in DVT patients. This finding is in accordance with previously published data from Kabrhel et al. [51], who reported a strong association between increasing body mass index and a higher rate of incident PE in a prospective cohort study of 87,226 women (Nurses’ Health Study). The authors outlined that the relative risk of unprovoked PE increased by approximately 8% per 1 kg/m^2^ higher BMI and approached an approximately six-fold elevated risk among individuals with a BMI ≥ 35 kg/m^2^ (*p* < 0.001) [51,52].

When comparing patients with isolated DVT (without concomitant PE) and isolated PE (without concomitant DVT), the prevalence of PAD was substantially higher in isolated DVT (without concomitant PE), whereas isolated PE (without concomitant DVT) occurred more often in old patients and those with chronic lung diseases and/or AF. These findings are in accordance with the literature, reporting that isolated PE (without concomitant DVT) was associated with high age and, in particular, with comorbid constitutions such as MI, AF and heart failure, therefore, underlining that the underlying pathomechanism of isolated PE (without concomitant DVT) might differ from the pathomechanism in PE with a causative DVT and travelled thrombotic material from peripheral veins to the pulmonary artery bed [17,20,21,23]. One other study showed that patients with isolated proximal DVT (without concomitant PE), as well as patients with DVT and PE, had an increased risk of death in comparison to controls, while patients with isolated PE without DVT had no decreased survival [53]. In the present study, we were not able to analyze the impact of different extents of DVT, such as calf, popliteal, femoral or iliac vein thrombosis on outcomes, since this information was not part of the assessed data. This has to be mentioned as a main limitation of our study. In this context, it is well known that the thrombus mass had an impact on study outcomes of VTE patients in recently published studies [19,54,55].

Subsuming our study results, we observed important differences in the atherosclerotic profile of DVT patients with and without PE, and therefore, the study provided some interesting insights regarding links between VTE and atherosclerosis. In particular, inflammation, hypercoagulability, platelet activation and also endothelial injuries are hypothesized to act as linking factors between atherosclerotic diseases and VTE [40,47,48,56]. In addition, elevated hemostatic markers typically attributed to VTE are also increased in patients with atherosclerosis, such as CAD and PAD [16,47,57,58]. Thus, these factors may contribute to thrombus formation in veins and arteries [40,47,48]. Chronic inflammation induces vessel wall alterations and is also associated with an increased risk for VTE [59,60,61,62]. In addition, it is well known that diabetes mellitus leads to macro- and microvascular defects followed by an acceleration of atherosclerosis. In addition, patients with diabetes mellitus have an imbalance of pro- versus anti-coagulants accompanied by an acquired thrombophilia. In particular, the non-enzymatic glycosylation of clotting inhibitors, such as antithrombin, and increased numbers of circulating microparticles bearing endogenous pro-coagulant triggers, result in a hypercoagulable state [42,63]. Diabetes mellitus was identified as a thromboembolic risk factor [42,63,64,65,66], although the exact pathomechanism regarding the association between diabetes mellitus and VTE remains unclear [42,63].

Furthermore, it has to be hypothesized that venous thrombus formation might differ in DVT patients with concomitant atherosclerotic diseases compared to those without atherosclerosis. The pathogenesis of arterial thrombosis formation is complex. Atherosclerosis leads to the detectable activation of platelets and blood coagulation, is related to inflammation and increases the fibrin turnover resulting in a higher frequency of thrombotic complications [16,47]. In contrast to the development of venous thrombi, the formation of arterial thrombi occurs despite higher blood flow [67]. Atherothrombosis arises more commonly on the basis of an atherosclerotic plaque [67,68], and thrombus formation in the arteries is more often found in vessels with pre-existing atherosclerotic lesions and stenoses [69,70]. Remarkably, after vein graft bypass surgery in CAD, atherogenesis is also observable in the vein grafts and seems to be accelerated. In comparison to the atherothrombosis in native coronary arteries, plaques in vein grafts are larger and contain more thrombus material [71].

In the present study, symptomatic atherosclerosis affected mortality and thromboembolic events during 2-year and 3-year follow-up significantly. These results for long-term follow-up are in accordance with previously published studies analyzing short-term outcomes of patients with PE and DVT [28,31]. Symptomatic atherosclerosis was associated with in-hospital case-fatality rate and adverse outcomes in patients with PE [31]. In addition, symptomatic atherosclerosis in DVT patients was accompanied by poorer in-hospital outcomes, including in-hospital death, and associated with higher bleeding rate and with isolated DVT (without concomitant PE) [28].

Although the link between VTE and atherosclerosis is recognized and established [13,16,26,28,31,39,40,41,42,43,44,45,46,47,48], consequences regarding screening for atherosclerotic diseases in cases of VTE events are rarely drawn. The present study results support the recommendation that in case of VTE events, regardless of whether DVT or PE entity is present, patients should be screened for concomitant (undetected) atherosclerotic disease.

## 5. Limitations

There are certain limitations of our study, which need to be mentioned: first, the study might be underpowered to identify associations between symptomatic atherosclerosis and VTE recurrence in the prospective 2-year and 3-year follow-ups. Nevertheless, this is an epidemiological study helping to generate hypotheses, but further basic scientific research on thrombus formation in the venous system under the influence of atherosclerosis is required in order to confirm the hypothesis of this study. As aforementioned, while previously published nationwide studies demonstrated an impact of atherosclerosis on short-term outcomes of patients with PE and DVT [28,31], the present study confirmed these important results regarding aggravating outcomes in DVT patients during 2-year and 3-year follow-up.

Second, since the included patients of the ThrombEVAL study were recruited from a mid-western European population in Germany of predominantly white European ancestry and the German health care system might differ from that of other countries, the results of the present study could only in part be generalized to other countries and populations.

Third, as we did not assess the extent of the DVT, we were not able to analyze the impact of different extents of DVT, such as calf, popliteal, femoral or iliac vein thrombosis, on outcomes.

Fourth, the study was widely conducted in the pre-novel oral anticoagulants (NOACs) era, and it is speculative whether the NOACs would influence the reported results.

## 6. Conclusions

Atherosclerosis was associated with isolated DVT (without concomitant PE) and increased mortality in DVT patients under oral anticoagulation. The profile of CVRF and comorbidities differed significantly between individuals with isolated DVT (without concomitant PE) and those DVT patients with concomitant history of PE. In the case of DVT or PE, patients should be screened for concomitant atherosclerotic disease.

## Figures and Tables

**Table 1:** Characteristics of patients with a history of deep venous thrombosis stratified for the presence of a concomitant pulmonary embolism.

	DVT without PE (*n* = 305)	DVT with PE (*n* = 204)	*p*-Value
Age (years)	70.0 (57.0/78.3)	70.0 (54.0/77.0)	0.49
Sex (Male)	145 (47.5%)	100 (49.0%)	0.79
**Classical cardiovascular risk factors**			
Obesity *	96 (31.5%)	77 (37.7%)	0.15
Diabetes mellitus	86 (28.2%)	33 (16.3%)	**<0.01**
Arterial hypertension	205 (67.2%)	124 (60.8%)	0.16
Dyslipidemia	134 (43.9%)	84 (41.2%)	0.58
Family history of myocardial infarction or stroke	126 (41.3%)	62 (30.4%)	**0.02**
Smoking (ex- or current smoker)	141 (46.2%)	84 (41.2%)	0.28
**Co-morbidities**			
Myocardial infarction	59 (19.4%)	24 (11.8%)	**0.03**
Coronary heart disease	94 (32.3%)	35 (17.8%)	**<0.001**
Congestive heart failure	87 (28.9%)	37 (18.2%)	**<0.01**
Atrial fibrillation	121 (40.1%)	47 (23.0%)	**<0.0001**
Peripheral artery disease	63 (21.3%)	22 (10.9%)	**<0.01**
Stroke	45 (14.8%)	21 (10.3%)	0.18
Chronic lung disease	59 (19.5%)	45 (22.3%)	0.05
Chronic kidney disease	60 (19.7%)	39 (19.2%)	0.91
Cancer	62 (21.0%)	38 (18.8%)	0.57
Depression	32 (10.5%)	21 (10.3%)	1.00
Symptomatic atherosclerosis ^†^	127 (42.9%)	52 (26.4%)	**<0.001**
Charlson comorbidity index ^§^	5.59 ± 3.10	4.93 ± 3.05	**0.02**

* Obesity was defined according to the World Health Organization (WHO, 2008) defining obesity as a BMI ≥ 30.0 kg/m^2^. ^†^ Symptomatic atherosclerosis was defined as the presence of coronary artery disease (CAD), myocardial infarction (MI) and/or peripheral artery disease (PAD). ^§^ The Charlson comorbidity index is a scoring system based on age, risk factors and comorbidities to evaluate the cormorbidity-burden and tp predict mortality in the future [37,38]. *p*-values < 0.05 were considered as significant associations.

**Table 2:** Characteristics of patients with a history of deep venous thrombosis (with and without PE) stratified for the presence of symptomatic atherosclerosis (defined as the presence of coronary artery disease, myocardial infarction and/or peripheral artery disease).

Variable	DVT without Symptomatic Atherosclerosis (*n* = 314)	DVT with Symptomatic Atherosclerosis (*n* = 179)	*p*-Value
Age (years)	63.0 (48.0–75.0)	74.0 (65.0–80.0)	**<0.0001**
Sex (Men)	138 (43.9%)	101 (56.4%)	**0.0087**
**Classical cardiovascular risk factors**			
Obesity *	95 (30.3%)	71 (39.7%)	**0.038**
Diabetes mellitus	47 (15.0%)	67 (37.4%)	**<0.0001**
Arterial Hypertension	173 (55.1%)	146 (81.6%)	**<0.0001**
Dyslipidemia	98 (31.2%)	115 (64.2%)	**<0.0001**
Family history of myocardial infarction or stroke	97 (30.9%)	89 (49.7%)	**<0.0001**
Smoking (ex- or current smoker)	118 (37.6%)	98 (54.7%)	**0.00024**
**Co-morbidities**			
Heart failure	45 (14.4%)	73 (41.2%)	**<0.0001**
Atrial fibrillation	73 (23.2%)	86 (48.3%)	**<0.0001**
Stroke	38 (12.1%)	26 (14.5%)	0.49
Chronic obstructive pulmonary disease	55 (17.5%)	47 (26.4%)	**0.021**
Chronic kidney disease	42 (13.4%)	54 (30.2%)	**<0.0001**
Cancer	62 (20.3%)	34 (19.3%)	0.81
Depression	33 (10.5%)	16 (8.9%)	0.64
Charlson comorbidity index	4.00 (2.00–6.00)	7.00 (5.00–8.00)	**<0.0001**

Abbreviations: DVT = deep venous thrombosis. * Obesity was defined according to the World Health Organization (WHO, 2008) defining obesity as a BMI ≥ 30.0 kg/m^2^. *p*-values < 0.05 were considered as significant associations.

**Table 3:** Impact of symptomatic atherosclerosis on outcomes of DVT patients regardless of the presence of additional PE during 2-year and 3-year follow-up periods under VKA treatment (*n* = 493 DVT patients; 24 months and 36 months follow-up periods): Univariate Cox regression.

	Univariable Analysis for 2-Year Follow-Up		Univariable Analysis for 3-Year Follow-Up	
Outcomes	HR (95% CI)	*p*-Value	HR (95% CI)	*p*-Value
All-cause mortality	1.98 (1.12–3.49)	**0.018**	2.34 (1.41–3.88)	**0.0010**
Major or clinically relevant bleeding	1.86 (0.96–3.63)	0.067	1.78 (1.00–3.17)	0.051
Hospitalizations	1.64 (1.21–2.21)	**0.0012**	1.70 (1.28–2.26)	**0.00023**
Primary long-term outcome	1.99 (1.31–3.04)	**0.0013**	2.00 (1.37–2.90)	**0.00030**
Thromboembolic arterial and venous events	2.13 (0.95–4.81)	0.068	2.36 (1.15–4.83)	**0.0019**
Recurrent venous thromboembolism	1.99 (0.36–10.88)	0.43	2.37 (0.69–8.13)	0.17

Abbreviations: HR = hazard ratio, CI = confidence interval. *p*-values < 0.05 were considered as significant associations.

## Data Availability

All authors take responsibility for all aspects of the reliability and freedom from bias of the data presented and their discussed interpretation.

## Supplementary Materials

The following supporting information can be downloaded at: https://www.mdpi.com/article/10.3390/life12050734/s1, Figure S1: Work flow of the telemedicine-based coagulation service [32,33,34,35]; Table S1: Inclusion and exclusion criteria of the thrombEVAL study [35]; Figure S2: A. Cardiovascular risk factors as independent predictors for isolated PE (without concomitant DVT) in multivari-able logistic regression models vs. patients with isolated DVT (without concomitant PE as the reference group): the model contained the following variables: sex, age, diabetes mellitus, obesity, hypertension, dyslipidemia, family history of myocardial infarction or stroke and smoking. B. Multivariable logistic regression models were used to evaluate the association with isolated PE (without concomitant DVT) (i.e., the dependent variable; reference: isolated DVT without concomitant PE). In model one, each concomitant disease was adjusted for cardiovascular risk factors (i.e., the independent variables) in a separate model and in model two, all concomitant diseases (myocardial infarction was not taken for adjustment due to the co-linearity with coronary artery disease and peripheral artery disease); Table S2: Characteristics of patients with a history of isolated deep venous thrombosis (without concomitant pulmonary embolism) vs. isolated pulmonary embolism (without concomitant deep venous thrombosis).

## Abbreviations

AF	Atrial fibrillation
CI	Confidence interval
CAD	Coronary artery disease
CKD	Chronic kidney disease
CLD	Chronic lung diseases
COPD	Chronic obstructive pulmonary disease
CS	Coagulation service
CVRF	Cardiovascular risk factors
DVT	Deep vein thrombosis
FH	Family history
HF	Congestive heart failure
MI	Myocardial infarction
OAC	Oral anticoagulant treatment
OR	Odds ratio
PAD	Peripheral artery disease
PE	Pulmonary embolism
RMC	Regular medical care
VKA	Vitamin K antagonist treatment
VTE	Venous thromboembolism
