# Supplementary material for: Atherosclerosis and Its Impact on the Outcomes of Patients with Deep Venous Thrombosis
Source: Life (Basel). 2022 May 14;12(5):734. doi: 10.3390/life12050734 (PMC9143312; doi:10.3390/life12050734)
Supplement: Supplementary file 1 [file life-12-00734-s001.zip › life-1718239-supplementary.pdf]

life

# Atherosclerosis and its impact on outcomes of patients with deep venous thrombosis

**Karsten Keller, MD[1,2,3], Jürgen H. Prochaska, MD[4,2,5], Meike Coldewey, MD[1,2], Sebastian Göbel, MD[1,5], Volker H. Schmitt, MD[1,5], Omar Hahad, PhD[1,5,6], Alexander Ullmann, DiplStat[2], Markus Nagler, DiplPsych[2], Heidrun Lamparter, DiplPsych[2], Christine Espinola-Klein, MD[1], Thomas Münzel, MD[1,5*], Philipp S. Wild, MD and MSc[4,5,2*]**

[1] Department of Cardiology, University Medical Center Mainz of Johannes Gutenberg-University Mainz, Mainz, Germany
[2] Center for Thrombosis and Hemostasis, University Medical Center Mainz of Johannes Gutenberg-University Mainz, Mainz, Germany
[3] Medical Clinic VII, Department of Sports Medicine, University Hospital Heidelberg, Heidelberg, Germany
[4] Preventive Cardiology and Preventive Medicine, Department of Cardiology, University Medical Center, Johannes Gutenberg University of Mainz, Mainz, Germany
[5] German Center for Cardiovascular Research (DZHK), Partner Site Rhein Main, Germany
[6] Leibniz Institute for Resilience Research (LIR) Mainz, Germany
*both authors contributed equally and should be considered as co-shared last authors.

**SUPPLEMENTAL APPENDIX**

# Atherosclerosis and its impact on outcomes of patients with deep venous thrombosis

**Keller et al.: DVT and atherosclerosis.**

Karsten Keller, MD[1,2,3], Jürgen H. Prochaska, MD[4,2,5], Meike Coldewey, MD[1,2], Sebastian Göbel, MD[1,5], Volker H. Schmitt, MD[1,5], Omar Hahad, PhD[1,5,6], Alexander Ullmann, DiplStat[2], Markus Nagler, DiplPsych[2], Heidrun Lamparter, DiplPsych[2], Christine Espinola-Klein, MD[1], Thomas Münzel, MD[1,5*], Philipp S. Wild, MD, MSc[4,5,2*]

[1] Department of Cardiology, Cardiology I, University Medical Center Mainz of Johannes Gutenberg-University Mainz, Mainz, Germany

[2] Center for Thrombosis and Hemostasis, University Medical Center Mainz of Johannes Gutenberg-University Mainz, Mainz, Germany

[3] Medical Clinic VII, Department of Sports Medicine, University Hospital Heidelberg, Heidelberg, Germany

[4] Preventive Cardiology and Preventive Medicine, Department of Cardiology, University Medical Center, Johannes Gutenberg University of Mainz, Mainz, Germany

[5] German Center for Cardiovascular Research (DZHK), Partner Site Rhein Main, Germany

[6] Leibniz Institute for Resilience Research (LIR) Mainz, Germany

* both authors contributed equally and should be considered as co-shared last authors

**Supplemental Methods**

*Study design and study sample*

In the RMC cohort, patients' VKA-treatment-management was continued by the physicians in charge (i.e. general practitioner, but also specialists) after the study enrolment [32-35]. Since the ThrombEVAL study is an observational study, treatment decisions were left to the discretion of the treating physicians and were not influenced by the study protocol or the investigators [32-35]. The physicians take the blood samples ambulatory during standard visits at the doctor's office of the RMC patients [32-35]. Blood samples were analysed in the laboratories, which were cooperating with the respective physician. Afterwards, depending the results of the laboratory tests, the dosing was performed by the physician contacting the patient [32-35].

The CS was operated by the Center of Thrombosis and Hemostasis, University Medical Center Mainz (Germany) (**Figure S1 in the supplemental material**) [32-35]. Specialized staff consisting of physicians and nurses of the CS, who were trained in hemostasis, were responsible for patients' VKA-treatment adjustments [32-35]. Standardized visits for anticoagulation control during fixed consultation hours at 16 urban and rural service points were offered and carried out. Additionally, the specialized CS staff performed home visits for frail and disabled patients [32-35]. The VKA treatment-relevant information assessed during the standardized assessment of clinical status at each visit including identification of complications as well as the level of international normalized ratio (INR) were recorded digitally in a web-based electronic patient file (Portavita B.V., Amsterdam, the Netherlands) [32-35]. Patients' obtained blood samples were analyzed in a central reference laboratory at the university medical center Mainz (Germany) facilitating comparable INR values [32-35]. The CS staff performed individual VKA dosing by incorporation and implementation of all relevant information in the electronic patient-file, supported by computer-assisted VKA dosing algorithms and subsequent professional individual optimization, if necessary [32-35]. The electronic patient-file was the interface for patient management in collaboration with general practitioners, specialists and

health care institutions [32-35]. Patients were contacted by telephone if their INR-values deviated from the INR target range for precise management recommendations given by the CS [32-35].

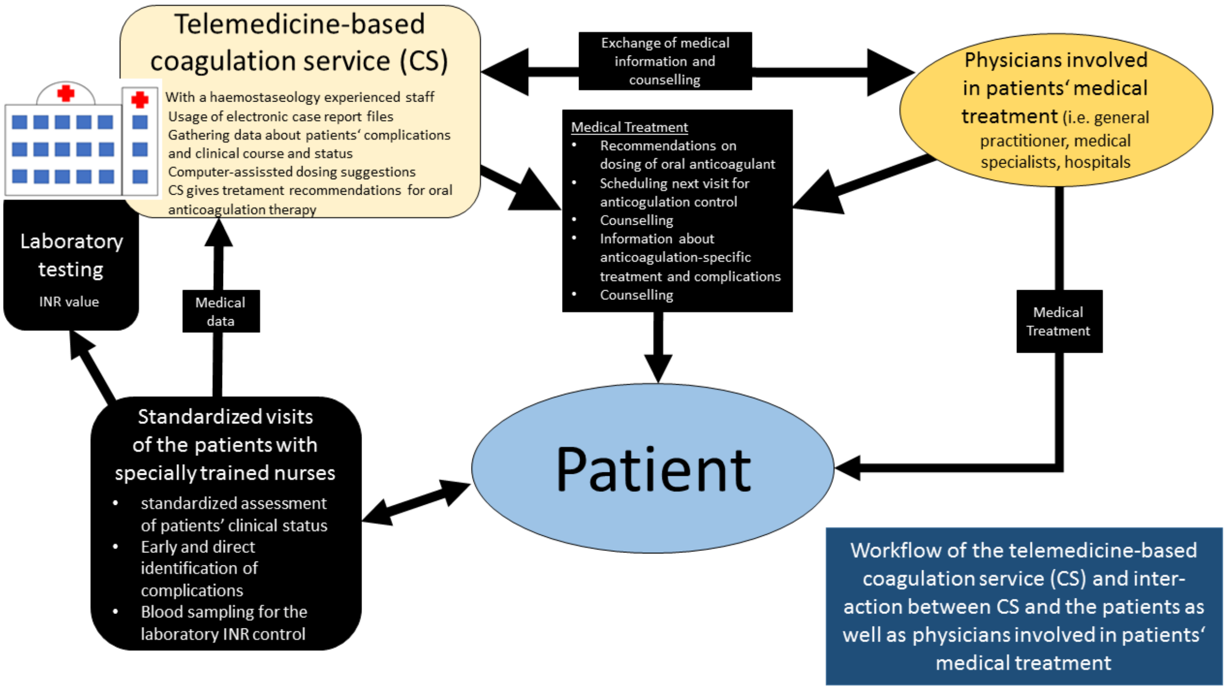


**Figure S1**: Work flow of the telemedicine-based coagulation service [32-35].

*Study definitions*

Classical cardiovascular risk factors comprised obesity (defined according to the World Health Organization (WHO, 2008) as a body mass index ≥30.0 kg/m2.), diabetes mellitus, arterial hypertension, dyslipidemia, family history of myocardial infarction or stroke and smoking. As important comorbidities MI, CAD, congestive heart failure, AF, PAD, stroke, chronic lung disease, chronic kidney disease, cancer and depression were assessed. In addition, the Charlson-Comorbidity-Index as a scoring system based on age, risk factors and comorbidities to evaluate the comorbidity-burden and to predict mortality in the future was assessed [37-38].

**Table S1:** Inclusion and exclusion criteria of the thrombEVAL study [35]

| **Inclusion criteria** | | **Exclusion crieria** |
|---|---|---|
| **Regular medical care** | **Coagulation service** | |
| Age ≥18 years | | Age <18 years |
| Written informed consent | | Withdrawal of priorly given consent |
| Performance of VKA therapy ≥4 months | | Contraindications for VKA treatment (e.g. pregnancy or known hypersensitivity) |
| | Indication for ≥3 months duration of VKA treatment | Participation in other clinical trials |

Abbreviations: VKA = vitamin K antagonist treatment

**Table S2:** Characteristics of patients with a history of isolated deep venous thrombosis (without concomitant pulmonary embolism) vs. isolated pulmonary embolism (without concomitant deep venous thrombosis)

| | Isolated DVT (n=305) | Isolated PE (n=91) | P-value |
|---|---|---|---|
| Age (years) | 70.0 (57.0/78.3) | 74.0 (68.0/80.0) | **0.0012** |
| Sex (Male) | 145 (47.5%) | 53 (58.2%) | 0.094 |
| **Classical cardiovascular risk factors** | | | |
| Obesity* | 96 (31.5%) | 28 (30.8%) | 1.00 |
| Diabetes mellitus | 86 (28.2%) | 19 (20.9%) | 0.18 |
| Arterial hypertension | 205 (67.2%) | 71 (78.0%) | 0.052 |
| Dyslipidemia | 134 (43.9%) | 37 (40.7%) | 0.63 |
| Family history of myocardial infarction or stroke | 126 (41.3%) | 27 (29.7%) | 0.050 |
| Smoking (ex- or current smoker) | 141 (46.2%) | 38 (41.8%) | 0.47 |
| **Co-morbidities** | | | |
| Myocardial infarction | 59 (19.4%) | 15 (16.9%) | 0.65 |
| Coronary heart disease | 94 (32.3%) | 37 (41.6%) | 0.13 |
| Congestive heart failure | 87 (28.9%) | 34 (38.2%) | 0.12 |
| Atrial fibrillation | 121 (40.1%) | 49 (53.8%) | **0.022** |
| Peripheral artery disease | 63 (21.3%) | 9 (10.2%) | **0.020** |

| | | | |
|---|---|---|---|
| Stroke | 45 (14.8%) | 8 (8.8%) | 0.16 |
| Chronic lung disease | 59 (19.5%) | 34 (37.8%) | **0.00063** |
| Chronic kidney disease | 60 (19.7%) | 20 (22.0%) | 0.66 |
| Cancer | 62 (21.0%) | 20 (22.2%) | 0.88 |
| Depression | 32 (10.5%) | 11 (12.2%) | 0.70 |
| Symptomatic atherosclerosis† | 127 (42.9%) | 40 (44.9%) | 0.81 |
| Charlson-Comorbidity-Index | 5.59±3.10 | 6.12±2.78 | 0.12 |

Abbreviations: DVT = Deep venous thrombosis; PE = pulmonary embolism

* Obesity was defined according to the World Health Organization (WHO, 2008) defining obesity as a BMI ≥30.0 kg/m$^2$

† Symptomatic atherosclerosis was defined as the presence of coronary artery disease (CAD), myocardial infarction (MI) and/or peripheral artery disease (PAD)

P-values <0.05 were considered as significant associations

**Figure S2:**

**A.** Cardiovascular risk factors as independent predictors for isolated PE (without concomitant DVT) in multivariable logistic regression models vs. patients with isolated DVT (without concomitant PE as the reference group): the model contained the following variables: sex, age, diabetes mellitus, obesity, hypertension, dyslipidemia, family history of myocardial infarction or stroke and smoking

**B.** Multivariable logistic regression models were used to evaluate the association with isolated PE (without concomitant DVT) (i.e., the dependent variable; reference: isolated DVT without concomitant PE). In model one, each concomitant disease was adjusted for cardiovascular risk factors (i.e., the independent variables) in a separate model and in model two, all concomitant diseases (myocardial infarction was not taken for adjustment due to the co-linearity with coronary artery disease and peripheral artery disease).

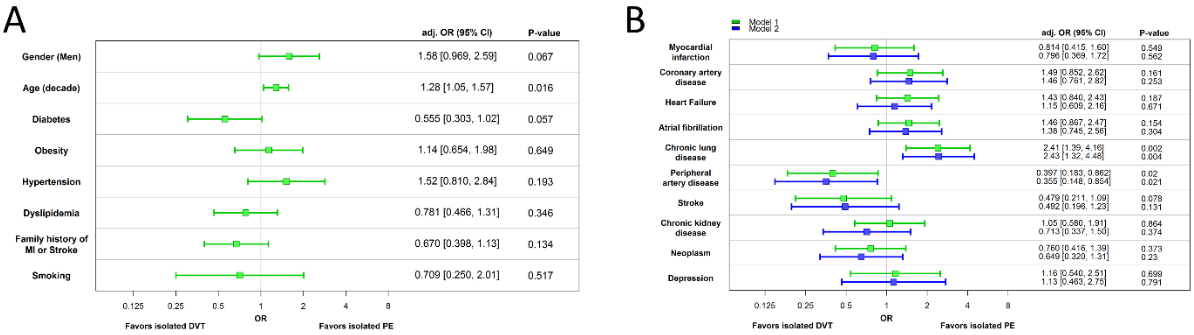


Abbreviations: DVT= deep venous thrombosis; PE= pulmonary embolism

P-values <0.05 were considered as significant associations

**Supplemental Text.**

**List of Study Investigators** (representatives of the study centers in alphabetic order).

Bickel C, MD, Kowitz C, Schmidt-Borko KM (Koblenz; Germany); Elsner M, MD, Hampel C, MD (Bad Kreuznach, Germany); Brako M, MD, Nietsch M, MD (Bad Kreuznach, Germany); Münchbach M, MD, Schwacke H, MD (Speyer, Germany); Meier-Willersen H, MD, Weis-Reinecke C, MD (Speyer, Germany); Heckmann J, MD (Bad Münster am Stein, Germany); Keck M, MD (Bad Münster am Stein, Germany); Schmidt W, MD (Bingen, Germany); Efferenn C, MD, Kratz T, MD (Simmern, Germany); Hardt R, MD, Wiechelt J, MD (Mainz, Germany), Jung, M, MD, Remplewski K, MD (Mainz, Germany), Dippold W, MD, Mertens D, MD (Mainz, Germany); Coldewey M, MD, Göbel S, MD, Hendelmeier M, MD, Keller K, MD, Münzel T, MD, Prochaska J, MD, Wild PS, MD, MSc (Mainz, Germany); Hauptmann, KE, MD; Lauterbach M, MD, Ulrich E, MD (Trier, Germany); Elsner S, MD, Küpper J, MD (Andernach, Germany); Inselmann G, MD (Ludwigshafen, Germany); Raedle J, MD (Kaiserslautern, Germany); Baldzhiev V, MD, Zotz R, MD (Bitburg, Germany), Hügl B, MD (Neuwied, Germany); Stefan V, MD, von Korn, H, MD (Neustadt an der Weinstraße, Germany); Schürmeyer TH, MD (Trier, Germany).
